# Comprehensive Comparison between Empty Nest and Non-Empty Nest Elderly: A Cross-Sectional Study among Rural Populations in Northeast China

**DOI:** 10.3390/ijerph13090857

**Published:** 2016-08-27

**Authors:** Ye Chang, Xiaofan Guo, Liang Guo, Zhao Li, Hongmei Yang, Shasha Yu, Guozhe Sun, Yingxian Sun

**Affiliations:** Department of Cardiology, the First Hospital of China Medical University, 155 Nanjing North Street, Heping District, Shenyang 110001, China; chang.ye@stu.xjtu.edu.cn (Y.C.); guoxiaofan1986@foxmail.com (X.G.); 13654970960@126.com (L.G.); xi.aohan1989@163.com (Z.L.); eileen8222@163.com (H.Y.); yidasasa@foxmail.com (S.Y.); gzhsun66@163.com (G.S.)

**Keywords:** rural population, northeast China, empty-nest elderly

## Abstract

This study aimed to comprehensively compare the general characteristics, lifestyles, serum parameters, ultrasonic cardiogram (UCG) parameters, depression, quality of life, and various comorbidities between empty nest and non-empty nest elderly among rural populations in northeast China. This analysis was based on our previous study which was conducted from January 2012 to August 2013, using a multistage, stratified, random cluster sampling scheme. The final analyzed sample consisted of 3208 participants aged no less than 60 years, which was further classified into three groups: non-empty nest group, empty nest group (living as a couple), and empty nest group (living alone). More than half of the participants were empty nest elderly (60.5%). There were no significant statistical differences for serum parameters, UCG parameters, lifestyles, dietary pattern, and scores of Patient Health Questionnaire-9 (PHQ-9) and World Health Organization Quality of Life questionnaire, abbreviated version (WHOQOL-BREF) among the three groups. Empty nest elderly showed no more risk for comorbidities such as general obesity, abdominal obesity, hyperuricemia, hyperhomocysteinemia, diabetes, dyslipidemia, left atrial enlargement (LAE), and stroke. Our study indicated that empty nest elderly showed no more risk for depression, low quality of life and comorbidities such as general obesity, abdominal obesity, hyperuricemia, hyperhomocysteinemia, diabetes, dyslipidemia, LAE, and stroke among rural populations in northeast China.

## 1. Introduction

The increasing aging population is a global significant social problem in the 21st century [1]. In China, the percentage of people aged ≥60 years was 11% of the total population in 2005 [2]. The empty nest families, in which there is only an elderly couple or one old person, increase year by year. In 2014, one survey conducted by China National Committee on Ageing announced that empty-nesters accounted for 51.1% of the elderly in China [3]. It is estimated that the proportion of empty nest elderly households will reach 90% by 2030, while all our elderly families will be “of the empty nest” [4]. Empty nest is becoming the main family pattern in China. Because of the declining fertility rate, the trend of young people to live independently after marriage, and more frequent population flow, the number of empty nest elderly is increasing rapidly [5], especially in rural areas, with the accelerated process of urbanization, the imbalance of economic development between the inland and coastal region, and the flow of rural surplus labor to big and Eastern coastal cities [6]. 

The empty nest elderly are vulnerable to different disadvantageous situations and experience problems associated with old age, such as health problems and irreversible decrease in function capacity [1]. On the other hand, the empty nest elderly have to strive to cope with psychosocial problems including loneliness, anxiety disorders, and depression: the so-called “empty nest syndrome” [4,5,7,8,9,10,11]. Besides, an increasing number of studies carried out in China have demonstrated that the social supports [8,9,12], quality of life [8,13], family function [9,12], and quality of sleep [14] were poor in empty nest elderly. The physical, psychological, and social problems of the empty-nest elderly will be a critical issue in the near future. Up to date, there are many studies from eastern China [9,10,12,13], central China [4,8,11], western China [3,5] focusing on issues of empty nest elderly. However, there is no study about the empty nest elderly among rural population in northeast China, where there is unique natural and humane environment.

Liaoning province is a mountainous region, located in northeast China. In rural villages, the vast majority of the inhabitants are involved in noncommercial agriculture performed by manual labor, and the yearly per capita income is low. The migration accounted for only a small proportion, so rural populations have similar socioeconomic backgrounds, lifestyles, and dietary habits. People in these rural areas still retain traditional lifestyles, such as working from sunrise to sunset with intervening rest, and a traditional diet pattern consisting largely of grains and low in meat.

The aim of this cross-sectional study was to comprehensively compare the general characteristics, lifestyles, serum parameters, ultrasonic cardiogram (UCG) parameters, depression, quality of life, and various comorbidities between empty nest and non-empty nest elderly among rural populations in northeast China.

## 2. Materials and Methods

### 2.1. Study Population

This study was conducted in Liaoning Province, located in northeast China. From January 2012 to August 2013, a representative sample of individuals aged ≥35 years was selected to characterize the prevalence, incidence, and natural history of cardiovascular risk factors in rural areas of Liaoning Province. The study adopted a multistage, stratified, random cluster sampling scheme. In the first stage, three counties (Dawa, Zhangwu, and Liaoyang County) were selected randomly from the rural areas of Liaoning province. In the second stage, one town was randomly selected from each of the three counties. In the third stage, 26 rural villages in 3 towns were randomly selected for inclusion in the study. All eligible permanent residents aged ≥35 years (a total of 14,016 individuals) in each village were invited to participate in the study. Of those, 11,956 participants (i.e., response rate of 85.3%) agreed to participate and completed the present study. Finally, all permanent residents aged ≥60 years were included in this study, with a response rate of 90.3%. The study protocol was approved by the Ethics Committee of China Medical University (Shenyang, China, ethical approved project identification code: 2011-2-2), and all procedures were performed in accordance with good ethical standards. Written consent was obtained from all participants after they had been informed of the objectives, benefits, medical items, and confidentiality of personal information. If the participants were illiterate, written informed consent was obtained from their proxies. In the present study, for participants aged ≥60 years, we obtained written informed consent from the proxies of 508 participators. Furthermore, 69 participators living alone were illiterate and were asked to put their fingerprint on the informed consent.

### 2.2. Lifestyle Factors

Our survey was performed by cardiologists and trained nurses during a single visit at a clinic in each village. Information on covariates, such as age, gender, and lifestyle, were collected using a standard questionnaire by face-to-face interview. Prior to conducting the survey, all eligible investigators were invited to attend a training session which covered topics including the purpose of the study, how to administer the questionnaire, the standard method of measurement, the importance of standardization, and study procedures. After completing the training session, each potential investigator was required to obtain a perfect score on a training test if they were to participate in the study. Additionally, the investigators received further instructions and support during periods of data collection.

Empty nest was measured by the following questions: (1) “How many people were there in your house living together with you in the past one year?” If the answer was not zero, then we asked; (2) “Who were they, spouse, children, or others?” Elders who lived alone or with spouse only were defined as empty nest elders, while those who lived with children were defined as non-empty elders [10,11,12]. Each study participant’s race was classified as either Han or others (which included ethnic minorities such as Mongol and Manchu). Annual personal income was calculated as family income divided by the number of permanent residents in family and was classified as ≤5000 and >5000 CNY/year. Period of residence was categorized as <10 years and >10 years. Educational level was categorized as low (no schooling, incomplete primary education, and primary education) and middle-high (3 or 4 years of secondary education, college, and university education). Physical activity was classified into three groups using a detailed description of the methods presented elsewhere [15]. Briefly, participants were asked with a question: “Which type do you think your occupational physical activity belongs to?” Occupational physical activity was grouped into three categories: (1) low was defined as participants who reported light levels of occupational physical activity, such as the elderly, crippled, and paralyzed; (2) moderate was defined as participants who reported moderate occupational physical activity, such as driver and office worker; (3) high was defined as participants who reported high level of occupational physical activity, such as manual agricultural activities and mining. The questionnaire included items related to average consumption (grams per week) of several food items (including legumes, vegetables, fruits, fish, poultry, and salt intake). Healthy diet was originally defined using the following five components: (1) legumes and cereals as basic food; (2) ≥500 g fruits and vegetables daily; (3) <100 g red meat/day; (4) regular (in most weeks) intake of soybean products and/or unprocessed fish; and (5) preference for non-salty food, in accordance with the current “Dietary Guidelines for Chinese Residents” [16]. Diet-score was 5 if subjects met the five criteria. Self-reported salt intake per month was obtained from the questionnaire. All study participants were asked whether or not they were currently smokers or drinkers and were classified as never (never smoke/drink or quit >12 months before), former (quitting ≤12 months before), or current (current smoker/drinker).

### 2.3. Blood Pressure Measurements and Definition of Hypertension

Based on the recommended American Heart Association protocol, blood pressure (BP) was measured three times at 2 min intervals after at least 5 min of rest using a standardized automatic electronic sphygmomanometer (HEM-907; Omron; Kyoto, Japan). The participants were advised to avoid caffeinated beverages and exercise for at least 30 min before the measurement. During the measurement, each participant was seated with their tested arm supported at the level of the heart. The mean of three BP measurements was calculated and used in all analyses. According to the JNC-7 report [17], hypertension is defined as systolic blood pressure (SBP) ≥140 mmHg and/or diastolic blood pressure (DBP) ≥90 mmHg and/or use of antihypertensive medications.

### 2.4. Anthropometric Measurements

Weight and height were measured to the nearest 0.1 kg and 0.1 cm, respectively, with the participant in lightweight clothing and without shoes. Waist circumference (WC) was measured at the umbilicus using a non-elastic tape (to the nearest 0.1 cm), and with the participant in a standing posture at the end of a normal expiration. Body mass index (BMI) was calculated as the individual’s weight in kilograms divided by the square of the height in meters. General obesity was defined as BMI ≥ 28 kg/m^2^, and abdominal obesity was defined as WC ≥ 80 cm for females and WC ≥ 85 cm for males, according to the recommendation of the Working Group on Obesity in China [18].

### 2.5. Echocardiography Measurements

Echocardiograms were obtained using a commercially available Doppler echocardiograph (Vivid, GE Healthcare, Fairfield, CA, USA), with a 3.0 MHz transducer. Echocardiogram analyses and readings were performed by three doctors specialized in echocardiography. The parasternal acoustic window was used to record two-dimensional and M-mode images of the left ventricular (LV) internal diameter and wall thickness. The apical acoustic window was used to record 4- and 5-chamber images. Correct orientation of planes for imaging and Doppler recordings was verified using previously described procedures [19]. LV internal dimensions and interventricular septal thickness (IVST) and posterior wall thickness (PWT) were measured at end-diastole and end-systole according to American Society of Echocardiography recommendations [19,20]. 

Left ventricular mass was calculated according to the equation LVM (g) = 0.81 (1.04 × (LVED + IVS + PWT))^3^ − (LVED)^3^ + 0.06 [21]. Left ventricular mass index (LVMI) was calculated by dividing left ventricular mass (LVM) by height in meters^2.7^ to correct LVM for body size [22]. LVH was defined as the LV mass indexed for height^2.7^ > 46.7 g/m^2.7^ in women and > 49.2 g/m^2.7^ in men [20]. The relative wall thickness (RWT) was calculated as 2× the posterior wall thickness/LV internal diameter at end-diastole and considered increased if >0.43 [23]. LV geometry was assessed from the LV mass/height^2.7^ combined with the RWT [21,23]; patients with normal LV mass/height^2.7^ were grouped into normal geometry (RWT ≤ 0.43) or concentric remodeling patterns (RWT > 0.43), and patients with elevated LV mass/height^2.7^ were grouped into eccentric (RWT ≤ 0.43) or concentric LV hypertrophy patterns (RWT > 0.43). LA enlargement was defined as left atrial diameter (LAD) > 40 mm [24]. 

### 2.6. Serum Analysis

A fasting blood sample was collected from each participant in the morning after at least 12 h of fasting. Blood samples were obtained from an antecubital vein and collected in vacutainer tubes containing EDTA. Values for fasting plasma glucose (FPG), total cholesterol (TC), low-density lipoprotein cholesterol (LDL-C), high-density lipoprotein cholesterol (HDL-C), triglyceride (TG), uric acid, homocysteine and other routine blood biochemical indexes were obtained using an autoanalyzer. All laboratory equipment was calibrated, and blinded duplicate samples were used. Dyslipidemia was defined as having at least one of high TC, high LDL-C, low HDL-C, and high TG, according to the National Cholesterol Education Program—Third Adult Treatment Panel (ATP III) criteria [25]. High TC was defined as TC ≥ 6.21 mmol/L (240 mg/dL). Low HDL-C was defined as HDL-C < 1.03 mmol/L (40 mg/dL). High LDL-C was defined as LDL-C ≥ 4.16 mmol/L (160 mg/dL). High TG was defined as ≥2.26 mmol/L (200 mg/dL). Diabetes was diagnosed according to World Health Organization (WHO) criteria: FPG ≥ 7 mmol/L (126 mg/dL) and/or being treated for diabetes [26]. Hyperuricemia was defined as uric acid ≥ 416.0 μmol/L (7.0 mg/dL) in man and ≥357.0 μmol/L (6.0 mg/dL) in women [27]. Hyperhomocysteinemia was defined as homocysteine ≥ 15 μmol/L [28], according to clinical cutoffs of plasma homocysteine.

### 2.7. Depression and Quality of Life

Patient Health Questionnaire-9 scale (PHQ-9) was used to evaluate the depressive symptoms. A total score for the nine items in PHQ-9 can range from 0 to 27 [29]. In our study, a score of 5 represented the cut point for depressive symptom (a) [10] and score of 10 represented the cut point for depressive symptom (b) [29]. Quality of life was assessed using the World Health Organization Quality of Life questionnaire, abbreviated version (WHOQOL-BREF) [30].

### 2.8. Coronary Heart Disease (CHD) and Stroke

Incidents of self-reported or family-reported CHD and stroke were obtained from a questionnaire, and all participants who reported an incident of CHD or stroke were asked to review their medical records, including reports of brain imaging, such as CT or MRI scanning.

### 2.9. Statistical Analysis

Descriptive statistics were calculated for all the variables, including continuous variables (reported as mean values and standard deviations) and categorical variables (reported as numbers and percentages). Histograms were used to test normality of the data distribution and all continuous variables at baseline failed the normality test. Differences among the three groups of living arrangements (non-empty nest elderly, empty nest elderly living a couple, and empty nest elderly living alone) were compared using one-way analysis of variance (ANOVA) with post-hoc Tukey tests for continuous variables. Comparisons of categorical variables between groups were performed using the χ^2^ test. Multivariable logistic regression analyses were used to identify association between the three groups of living arrangements and various comorbidities, adjusted for age and sex. Analyses are presented as odds ratio (OR) (95% CI). All the statistical analyses were performed using SPSS version 17.0 software (SPSS Inc., Chicago, IL, USA), and *p* values less than 0.05 were considered to be statistically significant. 

## 3. Results

A total of 3208 individuals (1572 males and 1636 females) aged ≥60 years participated in the study, including 1268 non-empty nest elderly, 1669 empty nest elderly living as a couple, and 271 empty nest elderly living alone. The prevalence of empty nest elderly was 60.5%. Table 1 shows the clinical and demographic characteristics of the study population. The average age was 67.0 ± 5.8 years old. Overall, the education level and annual personal income were low in rural areas. A total of 517 (16.1%) participants were poor or low-income residents, which was confirmed by our government. The vast majority of the population was Han ethnicity. Almost all participants settled down more than 10 years ago. Indeed, in the visit we found that the migration accounted for only a small proportion and most of the participants had even lived here for generations. Overall, in the three groups, the level of DBP, TC, and TG, UCG indices such as IVST, PWT, RWT, and LVM/H^2.7^ did not have significant statistical differences. For the values of BMI, WC, SBP, LDL-C, HDL-C, and UCG indices—such as left ventricular end diastolic diameter (LVEDD), LVM, and LAD—did reach significant statistical differences, however, the actual values did not vary greatly. Multiple comparisons using the post-hoc Tukey tests did not show any significant changes for WC, SBP, homocysteine, and LAD between any two groups, when *p* was set at 0.017 (0.05/3). The results of post-hoc tests for height, HDL, LVEDD, BMI, and LDL were statistically significant: for height, HDL, and LVEDD, empty nest elderly living as a couple versus non-empty nest and empty nest living alone (*p* < 0.001); for BMI, empty nest living alone versus non-empty nest and empty nest living as a couple (*p* < 0.01); for LDL and uric acid, empty nest as a couple versus non-empty nest (*p* < 0.01).

Table 2 shows the lifestyles and comorbidities in the three groups. There were no significant statistical differences in the status of smoking and drinking among the three groups. Overall, the level of physical activity was low, especially in the group of empty nest living alone. Salt intake per day and diet score did not show significant statistical differences. As to comorbidities, the prevalence of general obesity, abdominal obesity, hyperuricemia, and hyperhomocysteinemia also did not show significant statistical differences. In the questionnaire, we asked the participants whether they were informed by doctors that they suffered from hypertension/diabetes/dyslipidemia, if yes, whether they took medicines to control the disease, if yes, whether related parameters such as BP and FPG were treated to goal. Based on these questions, we calculated the awareness rate, treatment rate, and control rate of hypertension/diabetes/dyslipidemia. At the same time, we calculated the prevalence of hypertension/diabetes/dyslipidemia based on serum analysis. Overall, the prevalence of hypertension/diabetes/dyslipidemia was 71.4%, 14.6%, and 40.3% respectively, among the elderly. Furthermore, the awareness rate, treatment rate, and control rate decreased gradually. However, besides the prevalence of hypertension, there were no significant statistical differences for the prevalence of diabetes/dyslipidemia and the awareness rate, treatment rate, and control rate of hypertension/diabetes/dyslipidemia among the three groups. Additionally, the trend of left atrial enlargement (LAE) and self-reported stroke also did not reach statistical differences among the three groups. The prevalence of left ventricular enlargement (LVE) and self-reported CHD were higher in the group of empty nest living alone.

Table 3 shows the distributions of WHOQOL-BREF and PHQ-9 scores in the three groups. For WHOQOL-BREF, the scores of general quality of life, general health, physical, psychological, social interaction, and environment were 3.2 ± 0.7, 3.3 ± 0.9, 14.9 ± 2.3, 14.4 ± 2.5, 14.5 ± 2.1, and 13.4 ± 2.1, respectively. After standardization, calculated by (mean score/the highest total score) × 100, the scores were 64, 66, 74.5, 72, 72.5, and 67, respectively. Furthermore, there were no significant statistical differences for the scores of WHOQOL-BREF among the three groups. For PHQ-9, the mean score was 3.2 ± 3.9, and the percentage was 69.9%, 19.3%, 4.5%, and 2.0% in score group 0–5, 6–9, 10–14, and 15–27, respectively. There were no significant statistical differences for the scores of PHQ-9 among the three groups. The prevalence of depression (a) (PHQ-9 scores ≥ 5) and depression (b) (PHQ-9 scores ≥ 10) was 25.8% and 6.5%, respectively. Additionally, there were no significant statistical differences among the three groups.

Table 4 showed multivariable logistic regression analyses on the association between the three groups of living arrangements and comorbidities. As shown above, almost all variables showed no significant statistical differences among the three groups, so we only adjusted age and sex in multivariable logistic regression analyses. Taking non-empty nest elderly as reference, empty nest elderly showed no higher risk of suffering from depression, dyslipidemia, diabetes, hypertension, hyperuricemia, hyperhomocysteinemia, general obesity, abdominal obesity, CHD, stroke, LAE, and LVE. All of the *p* values were >0.05.

## 4. Discussion

To our knowledge, this was the first study to comprehensively compare the general characteristics, lifestyles, serum parameters, UCG parameters, depression, quality of life, and various comorbidities between empty nest and non-empty nest elderly among rural populations in northeast China, where people still follow traditional lifestyles. Unexpectedly, the study found that almost all variables did not reach significant statistical differences among the three groups: non-empty nest elderly, empty nest elderly living as a couple, and empty nest elderly living alone.

With the development of economy and society, the number of empty nest elderly increases year by year. A previous study indicated that the proportion of empty nest elderly households will reach 90% by 2030 [4]. Previous studies mainly focused on the psychosocial problems the empty nest elderly had to strive to cope with, including loneliness, anxiety disorders, and depression: the so-called “empty nest syndrome” [4,5,7,8,9,10,11]. Empty nest syndrome mainly resulted from children’s departure from home [4,9,10,11,12]. As demonstrated in these studies, Chinese people put high hopes on the notion of raising children for the purpose of being looked after in old age, so the elderly have a strong emotional dependence on and high expectation of their children. Besides the children’s departure from home, other factors including lower income, less social support and more negative coping style, education level, gender, retirees, and living in the rural areas also made the empty nest elderly more prone to depression [4,5,10,11]. A meta-analysis including 10 studies from China indicated that the life quality of empty nesters was statistically significantly lower in the rural area group compared to the urban area group [1]. Indeed, with the accelerated process of urbanization, the imbalance of economic development between the inland and coastal region, and the flow of rural surplus labor to big and eastern coastal cities, the number of empty nest elders is increasing rapidly in the rural areas [6]. Accumulating evidence indicates that in the rural population, empty nest elderly have higher levels of depression [4,11] and loneliness [8,31], and lower levels of social support [4], subjective wellbeing [32], quality of life [8,13], and life satisfaction [8] compared to non-empty nest elderly. 

However, inconsistent with previous studies, our study found that no significant statistical differences existed in depression and quality of life among the three groups (non-empty nest elderly, empty nest elderly living as a couple, and empty nest elderly living alone). What is the reason? Our results showed that almost all participants settled down more than 10 years ago. Indeed, during the visit we found that the migration accounted for only a small proportion and most of the participants had even lived here for generations. After marriage, children would divide up family property and live apart. Generally, they would not move too far away, often to neighboring villages. So, though empty nest elderly did not live with their children in the same house, they could meet easily. On the other hand, for the generation who were born before 1960s, because there was no restriction of “one-child policy”, they often had many brothers and sisters. At that time in China, the economy and the traffic were so underdeveloped that they often stayed around after marriage. This kinship would compensate for loneliness due to children’s departure. Third, different from urban residents, the rural population had been getting along well with each other for decades. In leisure time, they often got together to take part in activities such as playing poker and mahjong. Therefore, empty nest elderly may not feel loneliness and depression. Furthermore, for adults aged ≥60 years who were born before 1950s, they might have experienced wars. After the foundation of New China, they might have experienced the “Great Chinese Famine” from 1958 to 1961, and then “the Great Proletarian Cultural Revolution” from 1966 to 1976. This may be called “time brand”. The elderly experienced so much suffering that they may feel great happiness in modern life, which may explain why the quality of life based on WHOQOL-BREF scores showed no significant statistical differences among the three groups. Besides, as demonstrated by another study conducted in northeast China, northeast part of China was one of the most advanced industrial bases in the 1930s. However, the economic development in northeast China lagged behind south China, because of the reform and economic policies in those coastal regions [33]. Indeed, Liaoning province ranked in the bottom according to gross domestic product (GDP) for two years in 2014 and 2015. The decline in economic growth in northeast China in turn slowed down the improvement of the people's lifestyle, civilization, health awareness, and behaviors [33]. The percentage of annual personal income >20,000 CNY/year was 1.4% in all and self-perceived income in the rich group accounted for 2.1%. In comparison, studies in south China found that an obviously higher proportion of rural population had high level personal income [5,8,11,32]. The low level of annual personal income in northeast China may narrow the gap between the rich and poor and make the prevalence of depression and quality of life have no statistical differences between the empty nest elderly and non-empty nest elderly.

Besides depression and quality of life, we also compared many comorbidities among the three groups (non-empty nest elderly, empty nest elderly living as a couple, and empty nest elderly living alone). It was surprising that the prevalence of general obesity, abdominal obesity, hyperuricemia, hyperhomocysteinemia, diabetes, dyslipidemia, LAE, and stroke did not show significant statistical differences among the three groups. Even for the awareness rate, treatment rate, and control rate of hypertension, diabetes, and dyslipidemia, there were no significant statistical differences among the three groups. The results of multiple logistic regression analysis also confirmed that empty nest elderly had no more risk for these comorbidities compared to non-empty nest elderly. The main reason may be that the lifestyles such as smoking, drinking, and dietary pattern were similar. Besides, a study from southwest China reported that the education level and family per capita income were positively related with the willingness-to-pay for general practitioners among empty-nesters [3]. Overall, the education level and annual personal income were low in rural areas of northeast China. Regardless, the presence of the offspring at home, the elderly subjects of this region would not have access to cures. This may be another reason for not having found differences for these comorbidities between empty-nesters and non-empty-nesters. Additionally, many researchers have pointed out that on one hand, the empty-nester had higher risk for endocrine disorders and immune dysfunction which could further cause various diseases (e.g., cardiovascular disease, cancer) [8,34,35]; on the other hand, the empty-nester had lower use rate of healthcare services than the non-empty-nester [8]. As such, empty-nester may have higher mortality [10] and this could introduce the possibility of survivor bias. Survivor bias may be another reason for not having found differences in almost all variables among the three groups in our study.

Overall, because of socioeconomic factors, similar historical experience, and traffic inconvenience in the mountainous area, the elderly in the rural areas of northeast China kept similar lifestyles. This may be the main reason that there were no statistical differences in serum parameters, UCG parameters, depression, quality of life, and various comorbidities between empty nest and non-empty nest elderly among rural populations in northeast China. Secondly, the low level of education and annual personal income may be another reason for not having found differences in almost all variables among the three groups in our study. However, in general, the health status in the elderly was not optimistic. As an example, the prevalence of hypertension/diabetes/dyslipidemia in elderly living in the rural areas of Liaoning Province was high, while the rates of awareness, treatment, and control were unacceptably low. Furthermore, the prevalence of depression (a) was 25.8% in our population, higher than 10.3% in Zhejiang [10], which took the same PHQ-9 questionnaire with the same cutoff value. Therefore, the public should pay more attention and take serious actions to ensure the social–economic conditions and physical and psychological health of the elderly.

### Limitations

Several limitations in this study should be noted. First, the generality of our results would be reduced by the location of sample size. Second, the present study was a cross-sectional design, which cannot ascertain the directionality of the observed associations. Third, some data collected under the guidance of the researcher, because of the low literacy level of some older people, might result in some bias. Fourth, survivor bias may exist in our study. Last, no data on drug therapy were collected in our study, which may make the conclusions somehow elusive. Therefore, the analysis of the results in the study should be reviewed cautiously.

## 5. Conclusions

The present study suggested that empty nest elderly suffered neither higher risk for depression nor lower quality of life than non-empty nest elderly among rural populations in northeast China. Furthermore, the prevalence of comorbidities such as general obesity, abdominal obesity, hyperuricemia, hyperhomocysteinemia, diabetes, dyslipidemia, LAE, and stroke did not show significant statistical differences among the three groups.

## Figures and Tables

**Table 1:** Baseline characteristics of study population.

Variables	Total (*N* = 3208)	Non-Empty Nest (*N* = 1268)	Empty Nest		*p*-Value
			Living as Couple (*n* = 1669)	Living Alone (*n* = 271)	
**Demographic characteristics**					
Age (years)	67.0 ± 5.8	66.9 ± 6.3	66.4 ± 5.1	70.5 ± 6.2	<0.001
Male (%)	1572 (49.0)	582 (45.9)	876 (52.5)	114 (42.1)	<0.001
**Education**					<0.01
Low	2405 (75.0)	977 (77.1)	1210 (72.5)	218 (80.4)	
Middle-high	803 (25)	291 (22.9)	459 (27.5)	53 (19.6)	
**Annual personal income (CNY/year)**					<0.05
≤5000	2041 (63.6)	862 (68.0)	996 (59.7)	183 (67.5)	
>5000	1167 (36.4)	406 (32.0)	673 (40.3)	88 (32.5)	
Poor or low-income residents	517 (16.1)	215 (17.0)	229 (13.7)	73 (26.9)	<0.001
**Self-perceived income**					<0.05
Poor	1489 (46.4)	536 (42.3)	826 (49.5)	127 (46.9)	
Moderate	1645 (51.3)	697 (55.0)	809 (48.5)	139 (51.3)	
Rich	66 (2.1)	31 (2.4)	31 (1.9)	4 (1.5)	
**Race**					
Han (%)	3086 (96.2)	1220 (96.2)	1610 (96.5)	256 (94.5)	N.S.
Others **^a^** (%)	122 (3.8)	48 (3.8)	59 (3.5)	15 (5.5)	
**Period of resident (years)**					N.S.
10	32 (1.0)	15 (1.2)	16 (1.0)	1 (0.4)	
>10	3138 (99.0)	1240 (98.8)	1635 (99.0)	263 (99.6)	
**Anthropometric measures**					
Height (m)	1.58 ± 0.08	1.58 ± 0.08	1.59 ± 0.08	1.56 ± 0.08	<0.001
BMI (kg/m^2^)	24.4 ± 3.7	24.4 ± 3.9	24.4 ± 3.7	23.7 ± 3.6	<0.01
WC (cm)	82.8 ± 10.2	82.5 ± 10.0	83.3 ± 10.2	82.0 ± 10.3	<0.05
**Measurement indicators**					
SBP (mmHg)	152.0 ± 24.6	152.7 ± 24.9	151.0 ± 24.2	155.0 ± 25.2	<0.05
DBP (mmHg)	82.0 ± 11.6	82.1 ± 11.8	82.2 ± 11.5	80.6 ± 11.5	N.S.
LDL-c (mmol/L)	3.1 ± 0.9	3.2 ± 0.9	3.0 ± 0.8	3.0 ± 0.8	<0.001
HDL-c (mmol/L)	1.4 ± 0.4	1.4 ± 0.4	1.4 ± 0.4	1.5 ± 0.4	<0.001
TG (mmol/L)	1.7 ± 1.5	1.7 ± 1.5	1.7 ± 1.5	1.6 ± 0.9	N.S.
TC (mmol/L)	5.5 ± 1.1	5.5 ± 1.2	5.4 ± 1.1	5.5 ± 1.1	N.S.
FPG (mmol/L)	6.1 ± 1.8	6.1 ± 1.8	6.2 ± 1.8	6.0 ± 1.7	N.S.
Uric acid (mg/dL)	297.7 ± 84.5	292.7 ± 84.4	301.7 ± 84.1	296.6 ± 86.5	<0.05
Homocysteine (mmol/L)	19.2 ± 12.8	19.5 ± 12.9	18.6 ± 11.7	20.9 ± 17.2	<0.05
**UCG indices**					
IVST (cm)	0.93 ± 0.32	0.90 ± 0.34	1.01 ± 0.09	0.93 ± 0.13	N.S.
LVEDD (cm)	4.71 ± 0.49	4.68 ± 0.48	4.74 ± 0.50	4.65 ± 0.50	<0.01
PWT (cm)	0.90 ± 0.32	0.93 ± 0.40	0.90 ± 0.29	0.91 ± 0.37	N.S.
RWT	0.39 ± 0.11	0.39 ± 0.11	0.39 ± 0.11	0.39 ± 0.08	N.S.
LVM (g)	144.80 ± 37.34	142.66 ± 37.67	146.60 ± 36.90	143.56 ± 38.08	<0.05
LVM/H^2.7^ (g/h^2.7^)	42.11 ± 11.08	41.87 ± 11.15	42.11 ± 10.98	43.18 ± 11.30	N.S.
LAD	3.42 ± 0.46	3.41 ± 0.45	3.44 ± 0.47	3.34 ± 0.43	<0.05

Abbreviations: BMI, body mass index; CNY, China Yuan (1 CNY = 0.154 dollar); DBP, diastolic blood pressure; FPG, fasting plasma glucose; HDL-C, high-density lipoprotein; IVST, interventricular septal thickness; LAD, left atrial diameter; LDL-C, low-density lipoprotein cholesterol; LVEDD, left ventricular end diastolic diameter; LVEF, left ventricular ejection fraction; LVH, left ventricle hypertrophy; LVM, left ventricular mass; N.S., no statistical differences; PWT, posterior wall thickness; RWT, relative wall thickness; SBP, systolic blood pressure; TC, total cholesterol; UCG, ultrasonic cardiogram; WC, waist circumference; **^a^** Including some ethnic minorities in China, such as Mongol and Manchu.

**Table 2:** Lifestyles and comorbidities among participants.

Variables	Total (*N* = 3208)	Non-Empty Nest (*N* = 1268)	Empty Nest		*p*-Value
			Living as Couple (*n* = 1669)	Living Alone (*n* = 271)	
**Drink**					N.S.
Current	641 (20.0)	249 (19.6)	348 (20.9)	44 (16.2)	
Former	130 (4.1)	47 (3.7)	72 (4.3)	11 (4.1)	
Never	2437 (76.0)	972 (76.7)	1249 (74.8)	216 (79.7)	
**Smoke**					N.S.
Current	1103 (34.4)	445 (35.9)	568 (35.9)	90 (36.6)	
Former	27 (0.8)	11 (0.9)	15 (0.9)	1 (0.4)	
Never	1938 (60.4)	782 (63.2)	1001 (63.2)	155 (63.0)	
**Physical activity**					<0.001
Low	1884 (58.7)	781 (61.6)	913 (54.7)	190 (70.1)	
Middle	555 (17.3)	189 (14.9)	311 (18.6)	55 (20.3)	
High	769 (24.0)	298 (23.5)	445 (26.7)	26 (9.6)	
**Salt intake**	6.7 ± 3.8	6.7 ± 3.8	6.6 ± 3.8	6.6 ± 3.8	N.S.
**Diet score**	2.1 ± 1.3	2.2 ± 1.1	2.1 ± 1.1	2.0 ± 1.2	N.S.
**Comorbidities**					
General obesity	510 (15.9)	209 (16.5)	270 (16.2)	31 (11.4)	N.S.
Abdominal obesity	1689 (52.6)	642 (50.6)	909 (54.5)	138 (50.9)	N.S.
Hyperuricemia	358 (11.2)	128 (10.1)	194 (11.6)	36 (13.3)	N.S.
Hyperhomocysteinemia	1164 (36.3)	407 (32.1)	634 (38.0)	123 (45.4)	N.S.
**Hypertension**					
Prevalence	2291 (71.4)	927 (73.1)	1156 (69.3)	208 (76.8)	<0.01
Awareness	2112 (65.8)	456 (36.0)	552 (33.1)	88 (32.5)	N.S.
Treatment	843 (26.3)	358 (28.2)	414 (24.8)	71 (26.2)	N.S.
Control	124 (3.9)	53 (4.2)	63 (3.8)	8 (3.0)	N.S.
**Diabetes**					
Prevalence	468 (14.6)	186 (14.7)	249 (14.9)	33 (12.2)	N.S.
Awareness	234 (7.3)	90 (7.1)	128 (7.7)	16 (5.9)	N.S.
Treatment	189 (5.9)	74 (5.8)	105 (6.3)	10 (3.7)	N.S.
Control	23 (0.7)	8 (0.6)	14 (0.8)	1 (0.4)	N.S.
**Dyslipidemia**					
Prevalence	1293 (40.3)	514 (40.5)	675 (40.4)	104 (38.4)	N.S.
Awareness	281 (8.8)	119 (9.4)	146 (8.7)	16 (5.9)	N.S.
Treatment	127 (4.0)	48 (3.8)	73 (4.4)	6 (2.2)	N.S.
Control	49 (1.5)	17 (1.3)	29 (1.7)	3 (1.1)	N.S.
CHD	491 (15.3)	217 (17.1)	220 (13.2)	54 (19.9)	<0.01
Stroke	369 (11.5)	152 (12.0)	194 (11.6)	23 (8.5)	N.S.
LAE	241 (7.5)	86 (6.8)	135 (8.1)	20 (7.4)	N.S.
LVE	340 (10.6)	134 (10.6)	172 (10.3)	34 (12.5)	<0.05

Abbreviations: CHD, coronary heart disease; LAE, left atrial enlargement; LVE, left ventricular enlargement; N.S., no statistical differences.

**Table 3:** Patient Health Questionnaire-9 scale and the World Health Organization Quality of Life questionnaire scores stratified by empty nest group and non-empty nest group.

Variables	Total (*N* = 3208)		Non-Empty Nest (*N* = 1268)		Empty Nest		Empty Nest		*p*-Value
					Living as a Couple (*n* = 1669)		Living Alone (*n* = 271)		
	Scores (Mean ± SD)	Standardized Scores ^a^	Scores (Mean ± SD)	Standardized Scores ^a^	Scores (Mean ± SD)	Standardized Scores ^a^	Scores (Mean ± SD)	Standardized Scores ^a^	
Scores of WHOQOL-BREF									
General quality of life	3.2 ± 0.7	64	3.2 ± 0.7	64	3.2 ± 0.7	64	3.2 ± 0.6	64	N.S.
General health	3.3 ± 0.9	66	3.3 ± 0.8	66	3.3 ± 0.9	66	3.4 ± 0.9	68	N.S.
Physical	14.9 ± 2.3	74.5	14.9 ± 2.4	74.5	14.9 ± 2.3	74.5	14.7 ± 2.2	73.5	N.S.
Psychological	14.4 ± 2.5	72	14.4 ± 2.5	72	14.4 ± 2.5	72	14.4 ± 2.3	72	N.S.
Social interaction	14.5 ± 2.1	72.5	14.5 ± 2.2	72.5	14.4 ± 2.1	72	14.6 ± 1.9	73	N.S.
Environment	13.4 ± 2.1	67	13.5 ± 2.2	67.5	13.4 ± 2.1	67	13.3 ± 2.0	66.5	N.S.
Scores of PHQ-9	3.2 ± 3.9		3.3 ± 3.9		3.1 ± 3.8		3.6 ± 4.5		N.S.
0–5	2241 (69.9)		869 (70.7)		1172 (70.2)		173 (63.8)		N.S.
6–9	619 (19.3)		254 (20.0)		314 (18.8)		51 (18.8)		
10–14	145 (4.5)		65 (5.1)		68 (4.1)		12 (4.4)		
15–27	63 (2.0)		23 (1.8)		30 (1.8)		10 (3.7)		
Depression **^b^**	827 (25.8)		342 (27.0)		412 (26.0)		73 (29.7)		N.S.
Depression **^c^**	208 (6.5)		88 (6.9)		98 (5.9)		22 (8.1)		N.S.

Abbreviations: PHQ-9, Patient Health Questionnaire-9; WHOQOL-BREF, World Health Organization Quality of Life questionnaire; N.S., no statistical differences. **^a^** Standardized score = (mean score/the highest total score) × 100; **^b^** score of 5 represented cut point for depressive symptom; **^c^** score of 10 represented cut point for depressive symptom.

**Table 4:** Multivariable logistic regression analyses on the association between the three groups of living arrangement and comorbidities **^a^**.

**Empty Nest**	**Depression**			**Dyslipidemia**			**Diabetes**			**Hypertension**		
	**OR**	**95% CI**	***p*-Value**	**OR**	**95% CI**	***p*-Value**	**OR**	**95% CI**	***p*-Value**	**OR**	**95% CI**	***p*-Value**
Non-empty nest (reference)	1.00			1.00			1.00			1.00		
Empty nest living as a couple	0.94	0.79–1.11	N.S.	1.03	0.89–1.20	N.S.	1.05	0.85–1.29	N.S.	0.94	0.81–1.09	N.S.
Empty nest living alone	1.03	0.76–1.40	N.S.	0.93	0.71–1.23	N.S.	0.79	0.53–1.18	N.S.	1.06	0.78–1.46	N.S.
**Empty Nest**	**Hyperuricemia**			**Hyperhomocysteinemia**			**General Obesity**			**Abdominal Obesity**		
	**OR**	**95% CI**	***p*-Value**	**OR**	**95% CI**	***p*-Value**	**OR**	**95% CI**	***p*-Value**	**OR**	**95% CI**	***p*-Value**
Non-empty nest (reference)	1.00			1.00			1.00			1.00		
Empty nest living as a couple	1.17	0.92–1.48	N.S.	0.89	0.73–1.09	N.S.	0.99	0.81–1.21	N.S.	1.13	0.96–1.33	N.S.
Empty nest living alone	1.31	0.88–1.96	N.S.	0.78	0.56–1.09	N.S.	0.70	0.47–1.05	N.S.	0.97	0.74–1.27	N.S.
**Empty Nest**	**CHD**			**Stroke**			**LAE**			**LVE**		
	**OR**	**95% CI**	***p*-Value**	**OR**	**95% CI**	***p*-Value**	**OR**	**95% CI**	***p*-Value**	**OR**	**95% CI**	***p*-Value**
Non-empty nest (reference)	1.00			1.00			1.00			1.00		
Empty nest living with couple	0.88	0.73–1.05	N.S.	1.22	0.99–1.48	N.S.	1.15	0.87–1.53	N.S.	1.09	0.92–1.30	N.S.
Empty nest living alone	1.25	0.89–1.76	N.S.	1.03	0.72–1.48	N.S.	0.96	0.57–1.61	N.S.	1.22	0.91–1.64	N.S.

Abbreviations: CHD, coronary heart disease; LAE, left atrial enlargement; LVE, left ventricular enlargement; N.S., no statistical differences. **^a^** Adjusted for sex and age.

## References

[1] Lv X.L., Jiang Y.H., Sun Y.H., Ren C.Z., Sun C.Y., Sun L., Wu Z.Q., Zhao X. (2013). Short form 36-item health survey test result on the empty nest elderly in China: A meta-analysis. Arch. Gerontol. Geriatr..

[2] Liu L.J., Guo Q. (2008). Life satisfaction in a sample of empty-nest elderly: A survey in the rural area of a mountainous county in China. Qual. Life Res..

[3] Chen F., Xu X.L., Yang Z., Tan H.W., Zhang L. (2015). The willingness-to-pay for general practitioners in contractual service and influencing factors among empty nesters in Chongqing, China. Int. J. Environ. Res. Public Health.

[4] Su D., Wu X.N., Zhang Y.X., Li H.P., Wang W.L., Zhang J.P., Zhou L.S. (2012). Depression and social support between China rural and urban empty-nest elderly. Arch. Gerontol. Geriatr..

[5] Wang Z., Shu D., Dong B., Luo L., Hao Q. (2013). Anxiety disorders and its risk factors among the Sichuan empty-nest older adults: A cross-sectional study. Arch. Gerontol. Geriatr..

[6] Liu L.J., Sun X., Zhang C.L., Guo Q. (2007). Health-care utilization among empty-nesters in the rural area of a mountainous county in China. Public Health Rep..

[7] Fahrenberg B. (1986). Coping with the empty nest situation as a developmental task for the aging female—An analysis of the literature. Z. Gerontol..

[8] Liu L.J., Guo Q. (2007). Loneliness and health-related quality of life for the empty nest elderly in the rural area of a mountainous county in China. Qual. Life Res..

[9] Wang G., Zhang X., Wang K., Li Y., Shen Q., Ge X., Hang W. (2011). Loneliness among the rural older people in Anhui, China: Prevalence and associated factors. Int. J. Geriatr. Psychiatry.

[10] Zhai Y., Yi H., Shen W., Xiao Y., Fan H., He F., Li F., Wang X., Shang X., Lin J. (2015). Association of empty nest with depressive symptom in a Chinese elderly population: A cross-sectional study. J. Affect. Disord..

[11] Xie L.Q., Zhang J.P., Peng F., Jiao N.N. (2010). Prevalence and related influencing factors of depressive symptoms for empty-nest elderly living in the rural area of Yongzhou, China. Arch. Gerontol. Geriatr..

[12] Wu Z.Q., Sun L., Sun Y.H., Zhang X.J., Tao F.B., Cui G.H. (2010). Correlation between loneliness and social relationship among empty nest elderly in Anhui rural area, China. Aging Ment. Health.

[13] Liang Y., Wu W. (2014). Exploratory analysis of health-related quality of life among the empty-nest elderly in rural china: An empirical study in three economically developed cities in Eastern China. Health Qual. Life Outcomes.

[14] Li J., Yao Y.S., Dong Q., Dong Y.H., Liu J.J., Yang L.S., Huang F. (2013). Characterization and factors associated with sleep quality among rural elderly in China. Arch. Gerontol. Geriatr..

[15] Hu G., Tuomilehto J., Silventoinen K., Barengo N., Jousilahti P. (2004). Joint effects of physical activity, body mass index, waist circumference and waist-to-hip ratio with the risk of cardiovascular disease among middle-aged Finnish men and women. Eur. Heart J..

[16] Ge K. (2011). The transition of Chinese dietary guidelines and food guide pagoda. Asia Pac. J. Clin. Nutr..

[17] Chobanian A.V., Bakris G.L., Black H.R., Cushman W.C., Green L.A., Izzo J.L., Jones D.W., Materson B.J., Oparil S., Wright J.T. (2003). The seventh report of the joint national committee on prevention, detection, evaluation, and treatment of high blood pressure: The JNC 7 report. JAMA.

[18] Zhou B.F., Cooperative Meta-Analysis Group of the Working Group on Obesity in China (2002). Predictive values of body mass index and waist circumference for risk factors of certain related diseases in Chinese adults—Study on optimal cut-off points of body mass index and waist circumference in Chinese adults. Biomed. Environ. Sci..

[19] Sahn D.J., DeMaria A., Kisslo J., Weyman A. (1978). Recommendations regarding quantitation in m-mode echocardiography: Results of a survey of echocardiographic measurements. Circulation.

[20] Schiller N.B., Shah P.M., Crawford M., DeMaria A., Devereux R., Feigenbaum H., Gutgesell H., Reichek N., Sahn D., Schnittger I. (1989). Recommendations for quantitation of the left ventricle by two-dimensional echocardiography. American Society of Echocardiography Committee on standards, subcommittee on quantitation of two-dimensional echocardiograms. J. Am. Soc. Echocardiogr..

[21] Devereux R.B., Alonso D.R., Lutas E.M., Gottlieb G.J., Campo E., Sachs I., Reichek N. (1986). Echocardiographic assessment of left ventricular hypertrophy: Comparison to necropsy findings. Am. J. Cardiol..

[22] de Simone G., Daniels S.R., Devereux R.B., Meyer R.A., Roman M.J., de Divitiis O., Alderman M.H. (1992). Left ventricular mass and body size in normotensive children and adults: Assessment of allometric relations and impact of overweight. J. Am. Coll. Cardiol..

[23] Ganau A., Devereux R.B., Roman M.J., de Simone G., Pickering T.G., Saba P.S., Vargiu P., Simongini I., Laragh J.H. (1992). Patterns of left ventricular hypertrophy and geometric remodeling in essential hypertension. J. Am. Coll. Cardiol..

[24] Movahed M.R., Saito Y. (2008). Obesity is associated with left atrial enlargement, E/A reversal and left ventricular hypertrophy. Exp. Clin. Cardiol..

[25] Expert Panel on Detection, Evaluation, Treatment of High Blood Cholesterol in Adults (2001). Executive summary of the third report of the National Cholesterol Education Program (NCEP) Expert Panel on Detection, Evaluation, and Treatment of High Blood Cholesterol in Adults (Adult Treatment Panel III). JAMA.

[26] Yadav D., Mahajan S., Subramanian S.K., Bisen P.S., Chung C.H., Prasad G.B. (2013). Prevalence of metabolic syndrome in type 2 diabetes mellitus using NCEP-ATPIII, IDF and WHO definition and its agreement in Gwalior Chambal region of Central India. Glob. J. Health Sci..

[27] Fang J., Alderman M.H. (2000). Serum uric acid and cardiovascular mortality the NHANES I epidemiologic follow-up study, 1971–1992. National Health and Nutrition Examination Survey. JAMA.

[28] Kang S.S., Wong P.W., Malinow M.R. (1992). Hyperhomocyst(e)inemia as a risk factor for occlusive vascular disease. Annu. Rev. Nutr..

[29] Spitzer R.L., Kroenke K., Williams J.B. (1999). Validation and utility of a self-report version of PRIME-MD: The PHQ primary care study. Primary care evaluation of mental disorders. Patient health questionnaire. JAMA.

[30] Skevington S.M., Lotfy M., O’Connell K.A., Group W. (2004). The World Health Organization’s WHOQOL-BREF quality of life assessment: Psychometric properties and results of the international field trial. A report from the WHOQOL group. Qual. Life Res..

[31] Cheng P., Jin Y., Sun H., Tang Z., Zhang C., Chen Y., Zhang Q., Zhang Q., Huang F. (2015). Disparities in prevalence and risk indicators of loneliness between rural empty nest and non-empty nest older adults in Chizhou, China. Geriatr. Gerontol. Int..

[32] Zhou Y., Zhou L., Fu C., Wang Y., Liu Q., Wu H., Zhang R., Zheng L. (2015). Socio-economic factors related with the subjective well-being of the rural elderly people living independently in China. Int. J. Equity Health.

[33] Liu L., Zhang Y., Wu W., Cheng M., Li Y., Cheng R. (2013). Prevalence and correlates of dental caries in an elderly population in Northeast China. PLoS ONE.

[34] Zhou C., Ji C., Chu J., Medina A., Li C., Jiang S., Zheng W., Liu J., Rozelle S. (2015). Non-use of health care service among empty-nest elderly in Shandong, China: A cross-sectional study. BMC Health Serv. Res..

[35] Wang J., Zhao X. (2012). Empty nest syndrome in China. Int. J. Soc. Psychiatry.

